# Novel genetic risk factors influence progression of islet autoimmunity to type 1 diabetes

**DOI:** 10.1038/s41598-020-75690-6

**Published:** 2020-11-05

**Authors:** Suna Onengut-Gumuscu, Umadevi Paila, Wei-Min Chen, Aakrosh Ratan, Zhennan Zhu, Andrea K. Steck, Brigitte I. Frohnert, Kathleen C. Waugh, Bobbie-Jo M. Webb-Robertson, Jill M. Norris, Leslie A. Lange, Marian J. Rewers, Stephen S. Rich

**Affiliations:** 1 Center for Public Health Genomics, University of Virginia, PO Box 800717, Charlottesville, VA 22908 USA; 2 Barbara Davis Center for Childhood Diabetes, University of Colorado Anschutz Medical Campus, Aurora, CO USA; 3 Biological Sciences Division, Pacific Northwest National Laboratory, Richland, WA USA; 4 Colorado School of Public Health, University of Colorado Anschutz Medical Campus, Aurora, CO USA; 5 Department of Medicine, University of Colorado Denver, Anschutz Medical Campus, Aurora, CO USA

**Keywords:** Genome-wide association studies, Type 1 diabetes

## Abstract

Type 1 diabetes arises from the autoimmune destruction of insulin-producing beta-cells of the pancreas, resulting in dependence on exogenously administered insulin to maintain glucose homeostasis. In this study, our aim was to identify genetic risk factors that contribute to progression from islet autoimmunity to clinical type 1 diabetes. We analyzed 6.8 million variants derived from whole genome sequencing of 160 islet autoantibody positive subjects, including 87 who had progressed to type 1 diabetes. The Cox proportional-hazard model for survival analysis was used to identify genetic variants associated with progression. We identified one novel region, 20p12.1 (*TASP1*; genome-wide *P* < 5 × 10^–8^) and three regions, 1q21.3 (*MRPS21–PRPF3*), 2p25.2 (*NRIR*), 3q22.1 (*COL6A6*), with suggestive evidence of association (*P* < 8.5 × 10^–8^) with progression from islet autoimmunity to type 1 diabetes. Once islet autoimmunity is initiated, functional mapping identified two critical pathways, response to viral infections and interferon signaling, as contributing to disease progression. These results provide evidence that genetic pathways involved in progression from islet autoimmunity differ from those pathways identified once disease has been established. These results support the need for further investigation of genetic risk factors that modulate initiation and progression of subclinical disease to inform efforts in development of novel strategies for prediction and intervention of type 1 diabetes.

## Introduction

Type 1 diabetes is a complex autoimmune disorder whose etiology involves multiple genetic and environmental risk factors affecting up to 1 in 300 children^1^. The discovery of genetic variants associated with type 1 diabetes has accelerated greatly over the past years. Genome-wide association scan (GWAS) and fine-mapping efforts discovered over 40 risk loci^2,3^, with the majority of variants enriched in non-coding regions of the genome^3^.

The design of most genetic studies of type 1 diabetes typically involves comparison of cases (with varying duration of diabetes) with controls. The variants identified in such studies to be associated with type 1 diabetes reflect prevalent disease, and analyses thus preclude the factors that may be associated with the initiation of islet autoimmunity and the progression of subclinical disease^4^. The appearance of any of four beta-cell (islet) autoantibodies in the blood marks initiation of “islet autoimmunity” and is recognized as increasing risk for progression to type 1 diabetes^5,6^. The genetic contribution to initiation and progression of islet autoimmunity and clinical disease may be different (or overlapping), but this is not known.

The first region of the genome implicated in risk for type 1 diabetes, and the region with the greatest contribution to risk (~ one-half), includes the HLA genes on human chromosome 6p21^4,7^. Genetic variation in HLA-*DR* and HLA-*DQ* genes appears to be critical in expression of islet autoantibodies and progression/risk to type 1 diabetes, with contributions from other type 1 diabetes-associated genes (*PTPN22*, *UBASH3A*, *IFIH1, INS*, *PTPN2*)^8–11^; however, in these studies only a small subset of genetic variants (those most strongly associated with type 1 diabetes risk from case–control studies) have been examined, overlooking the vast majority of the human genome. Identifying genetic risk factors that play a role in the preclinical period of type 1 diabetes can help devise therapies aiming to stop the autoimmune process and preserve function of remaining beta-cells. This report aims to identify novel genetic factors that play a role in progression from islet autoimmunity to clinical type 1 diabetes.

## Results

### Association analysis for progression from islet autoimmunity to type 1 diabetes

A total of 160 participants in Diabetes AutoImmunity Study in the Young (DAISY)^12^, all of whom were persistently positive for islet autoantibodies, were characterized using whole genome sequencing. A total of 87 (54.4%) of these participants progressed to clinical type 1 diabetes during the follow-up period. The age at time of islet autoimmunity was younger (3.64 years) than those who did not progress (8.49 years, *P* < 0.0001). The duration of islet autoimmunity in those who progressed to type 1 diabetes was significantly shorter (6.48 years, *P* < 0.0001) than the follow-up period in those remaining disease free (10.89 years). Additional characteristics of the 160 DAISY participants are shown in Table 1.

**Table 1:** Demographics of 160 islet autoantibody positive DAISY participants.

	Progressors	Non-Progressors	*P*
	N = 87	N = 73	
**FDR, N (%)**	62 (71.26)	44 (60.27)	0.14*
**Female, N (%)**	45 (51.72)	38 (52.05)	0.97*
**NHW, N (%)**	79 (90.8)	50 (68.49)	0.0004*
**HLA DR 3/4, N (%)**	40 (45.98)	22 (30.14)	0.04*
**HLA DR, N (%)**			
3/4	40 (45.98)	22 (30.14)	0.047^†^
3/3 or 3/X	16 (18.39)	13 (17.81)	
4/4 or 4/X	26 (29.89)	25 (34.25)	
X/X	5 (5.75)	13 (17.81)	
**Age at seroconversion**			
Mean (SD)	3.64 (3.09)	8.49 (4.98)	< 0.0001^‡^
**Time from seroconversion**			
Mean (SD)	6.48 (4.73)	10.89 (5.89)	< 0.0001^‡^

Age at seroconversion: age of participant at time of seroconversion to persistent positivity for an autoantibody detected by either RBA or ECL methodology.

Time from seroconversion: time to T1D or last visit.

*FDR* first degree relative, *NHW* non-Hispanic white.

*Chi-square.

^†^Fisher’s exact.

^‡^Wilcoxon Rank Sum Test.

*p* < 0.05.

**Figure 1:**
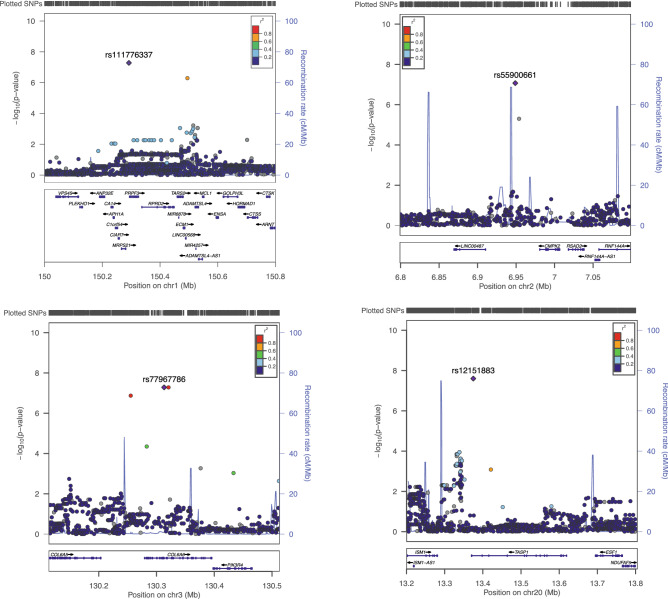
Regional association plots for 1q21.3, 2p25.2, 3q22.1 and 20p12.1. SNPs in each locus were plotted using LocusZoom^37^. The most significant SNP associated with progression to type 1 diabetes at each locus is plotted (purple). Each circle on the plot represents a single SNP included in whole genome sequencing association test, the symbol color corresponds to the degree of linkage disequilibrium with the most significant SNP, colored purple.

A total of 6,893,119 genetic variants (single nucleotide polymorphisms (SNPs) and small insertion or deletions (Indels)) were analyzed using the Cox proportional-hazard model for association with progression to type 1 diabetes. The genomic inflation factor (λ) compares the genome-wide distribution of the test statistic to that expected under the null distribution. Despite our relatively small population size, we observed λ = 1.03, supporting the absence of bulk inflation or excess false positive rate from the expected for this study. FFour independent regions in the genome showed evidence of association with progression to diabetes (Table 2, Fig. 1): one region attaining genome-wide significance, 20p12.1 (*TASP*; *P* < 5 x 10^–8^) and three regions with suggestive evidence (*P* < 8.5 × 10^–8^), 1q21.3 (*MRPS21*-*PRPF3*), 2p25.2 (*NRIR*), and 3q22.1 (*COL6A6*). None of these loci have been associated with type 1 diabetes in previous case–control analyses.

**Table 2:** SNPs associated with progression to type 1 diabetes.

Chr^a^	SNP	Position^b^ (bp)	Allele	AF^c^	HR (95% CI)	*P*	Gene^d^	Candidate gene^e^
1q21.3	rs111776337	150,319,828	T	0.050	6.4 (3.3–12.6)	5.23 × 10^–8^	*MRPS21, PRPF3*	*MCL1*
2p25.2	rs55900661	6,808,849	A	0.078	4.6 (2.6–8.1)	8.48 × 10^–8^	*NRIR*	*RSAD2*
3q22.1	rs77967786	130,594,375	A	0.056	5.5 (3.0–10.3)	5.19 × 10^–8^	*COL6A6*	
20p12.1	rs12151883	13,394,404	A	0.059	6.5 (3.4–12.8)	2.50 × 10^–8^	*TASP1*	*NDUFAF5*

^a^Chr: chromosome.

^b^Human genome assembly GRCh38/hg38.

^c^AF: allele frequency in DAISY cohort.

^d^Genes listed are based on proximity to lead SNP.

^e^Candidate genes identified based on functional annotation.

### Functional interpretation of genetic variants associated with progression

To gain insight into potential biological roles of the four loci (1q21.3, 2p25.2, 3q22.1 and 20p12.1) that showed evidence of association with progression to type 1 diabetes, we functionally annotated all SNPs with r^2^ > 0.6 (moderate linkage disequilibrium) with the lead SNP in each of the four novel regions, yielding 16 “credible SNPs” (a set of SNPs in each locus that have high probability of one being a causal variant, Supplementary Table 1). These SNPs were evaluated on their impact on gene function, gene expression and potential regulatory functions. Of the 16 credible SNPs, eleven were intergenic, four were intronic, and one resides in the 3′UTR sequence in *COL6A6*. Four of the SNPs were identified as cis-eQTLs (expression Quantitative Trait Loci, with the SNP-gene distance < 1 Mb).

In the 1q21.3 locus (*MRPS21-PRPF3*), the lead SNP rs111776337 is located in non-coding region of the genome and co-localizes with open chromatin landscape, in comparison to nearby SNP rs113588371 (r^2^ = 0.72), as seen in the majority of 127 tissue/cell types from ENCODE resource^13^. The location of the variant suggests a regulatory role in gene expression levels (eQTL). The eQTLGen Consortium database (https://www.eqtlgen.org/cis-eqtls.html) was queried for cis-eQTLs in whole blood. Although the nearest gene to rs111776337 is *PRPF3*, the variant is an eQTL for six genes: *MCL1, APH1A, CTSK, BNIPL, MRPS21, CDC42SE1* (Supplementary Table 2). With the lead SNP playing a role in expression levels of multiple genes in whole blood, we focused on traits associated to blood phenotypes in the UK Biobank (https://www.nealelab.is/uk-biobank/) to determine whether rs111776337 is associated with any immune system-relevant phenotypes. rs111776337 is most strongly associated with reduced monocyte count (*P* = 1.07 × 10^–8^) and reduced percentage of monocytes (*P* = 1.96 × 10^–8^).

In the 2p25.2 locus, the nearest gene to lead SNP rs55900661 is a long non-coding RNA that resides ~ 19 kb downstream; this gene is the negative regulator of interferon response (*NRIR*)*.* The functional annotations of eight credible SNPs in high linkage-disequilibrium (r^2^ > 0.6) suggests that rs55900661 is the strongest candidate, as it is the only SNP that is an eQTL to a gene immediately downstream of *NRIR*, radical S-adenosyl methionine domain containing 2 (*RSAD2*). *RSAD2* functions in interferon gamma signaling and toll like receptor signaling pathways, and is also an adaptor molecule that plays a role in CD4 + T-cell activation and differentiation^14^, making it a strong candidate for an autoimmune disease. rs55900661 and associated SNPs in 3q22.1 and 20p12.1, results discussed below, did not show any significant association with blood or immune phenotypes available in the UK Biobank.

In the 3q22.1 locus, the lead SNP, is located in the *COL6A6* gene (collagen, type VI, alpha 6 (uc003eni.4)). Alleles of this SNP do not affect expression levels of *COL6A6* or neighboring genes in blood. In GTEx data, rs77967786 is classified as a splice eQTL (ENSG00000206384.10) in pituitary tissue (*P* = 2.0 × 10^–33^).

In the 20p12.1 locus, the lead SNP, rs12151883, is located in the last intron of *TASP1* (Taspase 1). This gene functions in cleavage of the MLL protein, which is required for proper *HOX* gene expression. The rs2103987 credible SNP (r^2^ = 0.69 with lead SNP rs12151883) is an eQTL for Ubiquinone Oxidoreductase Complex Assembly Factor 5 (*NDUFAF5*).

## Discussion

This study is the first in-depth analyses of the potential role of genetic variants in progression from islet autoimmunity to clinical type 1 diabetes at the genome-wide level using whole genome sequencing. Our study identified four novel regions that have not been previously associated with type 1 diabetes risk in genome-wide association studies. Functional mapping of the associated SNPs indicates pathways critical to response to viral infections and response to interferon signaling are contributing to progression to type 1 diabetes.

The most promising gene associated with regulatory function of the lead SNP rs111776337 in the 1q21.3 locus is *MCL1*, a key anti-apoptotic protein in human beta-cells^15,16^. Apoptosis is one mechanism the host utilizes to eliminate virus-infected cells and is the main form of cell death in type 1 diabetes^17^. Reduced expression of MCL-1 has been observed in islets from patients with type 1 diabetes infected with a diabetogenic enterovirus, suggesting MCL-1 expression levels play a role in the development of diabetes in humans^18^.

The most promising gene associated with the lead SNP rs55900661 in the 2p25.2 locus appears to be *RSAD2*, also referred to as *VIPERIN* (virus inhibitory protein, endoplasmic reticulum–associated, interferon-inducible), can be induced by interferon and is known to play a role in immune response to DNA and RNA viruses, including human cytomegalovirus, which has been implicated as a potential trigger for type 1 diabetes^19^. In mice, rsad2 facilitates T-cell receptor-mediated GATA3 activation and optimal Th2 cytokine production by modulating NFKB1 and JUNB activities^14^. Transcript analyses in human islets indicate that expression of several genes connected to antiviral response increases including *IFIH1* (well established type 1 diabetes risk gene) and *RSAD2* in virus infected islet cells^20^. The role of *RSAD2* and progression to type 1 diabetes may be through its role in mounting an inflammatory response to viral infection of beta-cells.

*NDUFAF5*, is required for assembly of NADH-ubiquinone oxidoreductase complex (complex I) which is part of the mitochondrial respiratory chain that catalyzes the transfer of electrons from NADH to ubiquinone. To what extent metabolic dysregulation contributes to the breakdown of self-tolerance is still under investigation but there is evidence that mitochondrial metabolism plays an essential role for suppressive function of regulatory T-cells^21^.

This study has some limitations. First, the small sample size is underpowered to conduct the full-scale genome-wide analysis of variants contributing to progression of islet autoimmunity to diabetes and further limiting our ability follow-up findings focused on lead variants identified in case–control studies^2,3^. Steck et al.^11^, reported SNPs in *INS* (rs689, HR = 1.65, P = *0.03*), *UBASH3A* (rs11203203, HR = 1.44, *P* = 0.04) and *IFIH1* (rs1990760, HR = 1.47, *P* = 0.04) showed evidence of association with progression from islet autoimmunity to diabetes (*P* ≤ 0.04). While, these SNPs are not significant in our study, other than *INS* (HR = 1.00, *P* = 0.099), SNPs in *IFIH1* (HR = 1.26, *P* = 0.20) and *UBASH3A* (HR = 1.71, *P* = 0.20) do have effects in the same direction. Second, variant calls in evolutionary divergent regions of the genome, including HLA, have poor performance, possibly masking important roles of this complex in the etiology of the disease. A final limitation is the representativeness of the sample, as the DAISY participants are selected for increased type 1 diabetes risk based upon HLA genotype. While this sampling provides an accelerated transition from genetic risk to islet autoimmunity and diabetes, it does not mirror the distribution of HLA genotypes seen in the general population.

At the same time, there are important strengths of the study. This is the first application of whole genome sequence analysis to progression from islet autoimmunity to type 1 diabetes, generating several plausible candidate genes for inspection. Second, the DAISY cohort represents an important and well-characterized cohort of subjects followed longitudinally for development of islet autoantibodies and type 1 diabetes. An earlier analysis in the DAISY cohort has shown a slower progression to multiple islet antibodies and type 1 diabetes among participants that develop islet autoimmunity later in adolescence or early adulthood^22^. However, several studies have demonstrated that the development of multiple islet antibodies is strongly predictive of progression to type 1 diabetes^23–27^. Among the 107 DAISY participants diagnosed with type 1 diabetes to date, 7.5% were older than 20 years of age at the time of diagnosis (unpublished data). Continued follow-up of these young adults with persistent islet autoimmunity will help us to answer the question of what happens to the non-progressors as they age and will allow analysis of fast progressors versus slow progressors.

In summary, we identified four risk regions that may play a role in progression to clinical diabetes from islet autoimmunity. The most associated genes and variants identified here are not those that have been seen in previous case–control studies of type 1 diabetes, suggesting that the genetic impact on progression to diabetes from islet autoimmunity may differ in key pathways from those identified once disease is established and support the need for follow-up studies to understand genetic risk factors that modulate progression of subclinical disease.

## Methods

### Study population

The DAISY study has followed two cohorts of young children at increased risk of type 1 diabetes (total N = 2547): a cohort of relatives of type 1 diabetes patients (siblings and offspring) enrolled by age 7, and a general population newborn cohort. The latter consists of children with type 1 diabetes susceptibility HLA-DR/DQ genotypes identified through screening of over 31,000 newborns at St. Joseph Hospital in Denver, Colorado. The details of screening and follow-up have been previously published^28^. Islet autoimmunity is defined by persistence of autoantibodies to insulin, GAD65, IA-2 and ZnT8. These autoantibodies were measured in the Immunogenetic Laboratory at the Barbara Davis Center using radiobinding assays (RBA)^29^. Additionally, autoantibodies to insulin, GADA, and IA-2A were measured using electrochemiluminescent (ECL) assays in participants with islet autoantibodies detected by RBA^30,31^. The age at seroconversion was defined by the first appearance of at least one islet autoantibody detected by RBA or ECL that persisted for at least two consecutive visits. Duration of islet autoimmunity was calculated from age of seroconversion to age of type 1 diabetes diagnosis (or age at last visit). Islet autoantibodies were measured at 9, 15, and 24 months and annually thereafter; autoantibody positive children were tested every 3–6 months. Type 1 diabetes onset was defined according to ADA criteria. DAISY participants included in this analysis were enrolled into the follow-up study by 7 years of age (enrollment criteria for the first-degree relative cohort) and were shown to be persistently positive for one or more islet autoantibody by RBA or ECL assay. This subset of participants are described here as “progressors”-those that progressed to clinical diagnosis of type 1 diabetes- and “non-progressors”-those that had not been diagnosed at the time of the last clinic visit or the last direct contact. Non-progressors were censored at the time of last direct contact: in-person or by phone, email, or text.

Informed consent was obtained from the parents of each study subject. The Colorado Multiple Institutional Review Board approved all study protocols. All experimental methods were carried out in accordance with relevant guidelines and regulations.

### Whole genome sequencing

We sequenced 160 islet autoantibody positive DAISY participants on the Illumina HiSeq-X10 platform, yielding an average coverage of ~ 30-fold per base. Quality control checks were performed using FastQC^32^, and sequencing reads were aligned to the GRCh37 + decoy reference genome using BWA^33^ with default settings. We identified ~ 18.52 million SNPs and ~ 2.09 million indels using the reference model (gVCF-based) workflow for joint analysis using GATK-HaplotypeCaller^34^. Per sample variant calling metrics, determined using CollectVariantCallingMetrics tool, are provided in Supplementary Table 3.

### Statistical analyses

Prior to statistical analysis, we filtered out variants with minor allele frequency < 0.05, call rate < 90% and/or HWE *P* < 10^–10^ in northern European samples, 6,893,119 variants remained for statistical analysis. Principal components (PCs) of ancestry were generated using KING^35^. Among the 160 samples, 12 pairs of full-siblings were identified, and the familial correlations among siblings were adjusted using the frailty function in R^36^ which added a simple random effects term to the Cox proportional-hazard model. In Cox proportional-hazard model for survival analysis, the time from seroconversion to either date of diabetes diagnosis or time of last contact was the time-to-event variable, the genotype at each variant was the independent variable, and the covariates to be adjusted included sex, age at seroconversion, first four PCs of ancestry, and the HLA haplotype groups (defined by DR3/4 as shown in Table 1).

### Functional annotation

We conducted functional annotation using FUMA-v1.3.5e (https://fuma.ctglab.nl).

## Supplementary information

Supplementary Table 1.

Supplementary Table 2.

Supplementary Table 3.

## Data Availability

All data used in the development of this manuscript is being deposited into dbGaP.
